# Matrix Metalloproteinases and Bladder Cancer: What is New?

**DOI:** 10.5402/2012/581539

**Published:** 2012-07-17

**Authors:** O. Rodriguez Faba, J. Palou-Redorta, J. M. Fernández-Gómez, F. Algaba, N. Eiró, H. Villavicencio, F. J. Vizoso

**Affiliations:** ^1^Department of Urology, Universitat Autonòma de Barcelona, Barcelona, 08025 Fundació Puigvert, Spain; ^2^Department of Urology, Universidad de Oviedo, 33003 Oviedo, Spain; ^3^Department of Pathology, Universitat Autonòma de Barcelona, Barcelona, 08025 Fundació Puigvert, Spain; ^4^Unidad de Investigacion, Hospital de Jove, 33290 Gijón, Spain

## Abstract

Urothelial bladder cancer represents a heterogeneous disease with divergent pathways of tumorigenesis. Tumor invasion and progression are a multifactorial process promoted by microenvironmental changes that include overexpression of matrix metalloproteinases (MMPs). Recent data clearly challenge the classic dogma that MMPs promote metastasis only by modulating the remodeling of extracellular matrix. Indeed, MMPs have also been attributed as an impact on tumor cell behavior in vivo as a consequence of their ability to cleave growth factors, cell surface receptors, cell adhesion molecules, and chemokines/cytokines. Levels of the different MMPs can be measured in several sample types, including tissue, blood (serum and plasma), and urine, and using different methodologies, such as immunohistochemistry, real-time PCR, western and northern blot analyses, enzyme-linked immunosorbent assay, and zymography. Several MMPs have been identified as having potential diagnostic or prognostic utility, whether alone or in combination with cytology. Although MMP inhibitors have shown limited efficacy, advances in the understanding of the complex physiologic and pathologic roles of MMPs might permit the development of new MMP-specific and tumor-specific therapies. In this paper we update the understanding of MMPs based on a systematic PubMed search encompassing papers published up to December 2011.

## 1. Introduction

Urothelial bladder carcinoma (UBC) represents a heterogeneous disease with high morbidity and mortality. It ranks fifth among all cancers in the Western world, and there are 336,000 new cases and 132,000 deaths annually worldwide [1]. UBC mainly affects the elderly, with the peak incidence occurring after the age of 50. It is more common in males, in white people, and in developing countries. Most cases are sporadic, so a familial history is lacking. Cigarette smoking is a major risk factor. Other risk factors include exposure to arylamines, radiotherapy to adjacent organs, alkylating chemotherapeutic agents, and chronic inflammation [2]. There are two theories regarding the multifocal nature of urothelial carcinoma: “field cancerization” and “monoclonality” [3]. Genetic studies support the monoclonality theory and indicate that tumor cells spread from their origins to multiple sites by intraepithelial or intraluminal seeding. Non-muscle-invasive (NMI) disease accounts for ca. 70% of cases. Despite treatment with BCG, high-grade NMI tumors are associated with a high risk of recurrence and progression. Approximately 20% of primary diagnosed UBCs are muscle invasive. Platinum-based chemotherapy remains the treatment of first choice in neoadjuvant, adjuvant, and metastatic disease. However, ultimately disease recurs in most patients and life expectancy after recurrence is limited. Current clinical and pathologic predictive factors (stage, grade, associated carcinoma in situ (CIS), lymph node status, multiplicity, sex) are insufficient to predict prognosis [4]. Efforts are focusing on the study of the urothelial tumorigenesis and the development of new biomarkers with prognostic and predictive utility. Moreover, UBC is one of the few tumors where the direct administration of therapeutic drugs to the tumoral cells is feasible via the intravesical route, offering the maximum local drug concentration and minimum systemic toxicity.

## 2. Urothelial Tumorigenesis

Urothelial tumors arise and evolve through divergent phenotypic pathways. Less aggressive tumors range from urothelial hyperplasia to low-grade NMI papillary tumors. The latter low-grade variant is often multifocal and presents high rates of recurrence. More aggressive variants either arise from high-grade and flat lesions of CIS and progress to become invasive tumors or initially develop as invasive tumors. Despite BCG or cystectomy, metastases often develop [5].

The low-grade NMI variant is characterized by activation of mutations in the *HRAS* gene and the fibroblast growth factor receptor 3 gene [6]. High-grade and invasive tumors are characterized by structural and functional defects in the p53 and retinoblastoma protein (RB) tumor suppressor pathways [7]. Deletion of both arms of chromosome 9 is prevalent in urothelial carcinomas and occurs during the earliest stages of tumorigenesis; however, the genetic alterations in chromosome 9 do not seem to distinguish between the two tumor development pathways [8].

Tumor invasion and progression in the bladder represent a multifactorial process promoted by microenvironmental changes that include upregulation of N-cadherin, downregulation of E-cadherin, overexpression of matrix metalloproteinases (MMP) 2 and 9, imbalance between angiogenic and antiangiogenic factors, and increased synthesis of prostaglandin.

## 3. Matrix Metalloproteinases

Levels of the different MMPs can be measured in several sample types, including paraffin-embedded or fresh-frozen tissue, serum, plasma, and urine, and by various analytical methodologies, such as immunohistochemistry, real-time PCR, western and northern blot analyses, enzyme-linked immunosorbent assay, and zymography. Several MMPs have been identified as having potential diagnostic or prognostic utility, whether alone or in combination with currently available diagnostic tests or imaging modalities. Although the early broad-spectrum anti-MMP agents showed a lack of efficacy, our continually improving understanding of the complex physiologic and pathologic roles of MMPs might enable the development of new MMP-specific and tumor-specific therapies.

### 3.1. Classification and Structure

MMPs are a large family—there are at least 26 human MMPs—of calcium-dependent zinc-containing endopeptidases, which are responsible for the tissue remodeling and degradation of proteins of the extracellular matrix (ECM), including collagens, elastins, gelatin, matrix glycoproteins, and proteoglycans. On the basis of their specificity, MMPs are classified into collagenases, gelatinases, stromelysins, and matrilysins.

Another subclass of MMPs is represented by the membrane-type MMPs (MT-MMPs), which contain a transmembrane and an intracellular domain a membrane linker domain or are membrane associated [9] (Table 1).

The exact domain organization, polypeptide fold, and main specificity determinants are provided by the crystal structures of MMPs [10]. Most MMPs are composed of several distinct domains: an N-terminal prodomain (also termed propeptide), a catalytic domain (MMP-7 and MMP-26), and a hemopexin-like C-terminal domain connected by a linker or hinge region (MMP-1, MMP-3, MMP-8, MMP-11, MMP-12, MMP-13, MMP-18, MMP-19, MMP-20, MMP-21, MMP-27, and MMP-28). Others additionally have a fibronectin-like domain (MMP-2 and MMP-9), a transmembrane region (MMP-14, MMP-15, MMP-16, and MMP-24), or a glycosylphosphatidyl anchor (MMP-17 and MMP-25) [11]. The N-terminal prodomain contains in its motif PRCGXPD, a cysteine residue which interacts with the catalytic domain, preserving the MMP inactivity. The catalytic domain, sequence motif HEXGHXXGXXH, contains three histidine residues which ligate the zinc ion and the glutamic acid residue, facilitating catalysis. The catalytic domain additionally contains a conserved methionine, forming a “Met-turn” eight residues after the zinc binding motif, which forms a base to support the structure around the catalytic zinc. The zinc binding motif and the Met-turn are conserved in members of the ADAM (a disintegrin and metalloproteinase) family and the ADAMTS (ADAM with thrombospondin motifs) family [12].

### 3.2. Activation

MMPs can be activated in vitro by several mechanisms, including mercurial compounds, SH reagents, chaotropic agents, and other proteases [13]. This property is used to activate proMMPs in the laboratory. The N-terminal prodomain maintains MMPs in their zymogen form (proMMPs); these zymogens are then processed by other proteolytic enzymes, such as serine proteases or plasmin, to generate the active forms. Thirteen mammalian MMPs are secreted from the cell as proMMPs. The propeptide contains a “bait” region for proteinase that allows proMMP activation by tissue and plasma proteinases. Cleavage of the bait region removes only a part of the propeptide, and complete removal of the propeptide is often conducted in trans by the action of the MMP intermediate or by other active MMPs. This is the mechanism termed “stepwise activation” [14]. A furin-like proprotein convertase recognition sequence, RX[K/R]R, is present at the end of the propeptide in ten proMMPs. These proMMPs may be activated intracellularly, cell surface bound, or secreted [15].

### 3.3. Inhibitors

MMP activities are regulated by two major types of endogenous inhibitor: *α*_2_-macroglobulin and tissue inhibitors of MMPs (TIMPs). Human *α*_2_-macroglobulin is a plasma glycoprotein of 725 kDa consisting of four identical subunits of 180 kDa. It inhibits most proteinases by entrapping the proteinase within the macroglobulin, and the complex is rapidly cleared by receptor-mediated endocytosis [16]. TIMPs are a family comprising four members (TIMP-1, -2, -3, and -4). They consist of 184–194 amino acids subdivided into an N-terminal and a C-terminal subdomain.

Each domain contains three conserved disulfide bonds and the N-terminal domain folds as an independent unit with MMP-inhibitory activity. TIMPs inhibit all MMPs tested so far; however, TIMP-1 is a poor inhibitor for MT1-MMP, MT3-MMP, MT5-MMP, and MMP-19. TIMP-3 has been shown to inhibit members of both the ADAM family (ADAM-10, -12, and -17) and the ADAMTS family (ADAMTS-1, -4, and -5). In addition, TIMP-1 inhibits ADAM-10. Crystal structures of the TIMP-MMP complexes have been shown to be central to the mechanism of TIMP inhibition of MMPs [17, 18]. TIMPs have four N-terminal residues that are linked by a slot into the active site of the MMPs. This region accounts for about 75% of the protein–protein interaction in the case of the complex of the catalytic domain of MMP-3 and TIMP-1. TIMP concentrations generally far exceed the concentration of MMPs in tissue and extracellular fluids, thereby limiting the proteolytic activity of MMPs to focal pericellular sites. In contrast to the usual inhibitory role of TIMPs, a low concentration of TIMP-2 enhances MMP-14-induced activation of MMP-2 by forming a triplex with these proteins on the cell surface [19]. In addition, TIMPs have been shown to have growth-promoting activities which are independent of their MMP inhibitory function and apoptosis-inducing properties (TIMP3). The transcription of TIMPs is regulated by cytokines and growth factors similar to those that control MMP expression, although often in distinctive ways.

#### 3.3.1. S_1_ ****′ Specificity Loop Inhibitor

Differences in MMP active sites reside in specificity loop residues that form the S_1_′ subsite pocket. This leads to structural and chemical differences in the S_1_′ subsite that are reflected in the substrate preferences of the shallow pocket MMPs (1 and 7) compared with deep pocket MMPs (2, 3, 8, 9, 12, and 13) [20]. The development of novel specific inhibitors for MMP-12 and MMP-13 is attributable to differences in the S_1_′ pocket. An additional small region termed the “S_1_′ side-pocket” was used to specifically target MMP-13. The success of these inhibitors clearly demonstrates the benefits of good mechanistic and structural information for inhibitor design.

#### 3.3.2. Zinc-Binding Group Inhibitors

Zn^2+^-chelating hydroxamates have been favored in MMP inhibitor design. However, in some cases they overwhelm the contribution from the rest of the compound, reducing opportunities for improved specificity. Indeed, hydroxamate activity-based MMP probes bind many off-target metalloproteases outside the MMP family. The use of union to Zn^2+^ ion seems to require an improvement in specificity.

#### 3.3.3. Covalent Inhibitors

Upon binding, these inhibitors covalently modify residues in the active site of the enzyme. Since the reactive species is formed only within the active site of the targeted enzyme, it provides high specificity and in vivo selectivity. Thiirane sulfur-containing anti-MMP-2 and -9 inhibitor forms a reversible covalent bond with the active site glutamate and was found to exert a great effect in an aggressive murine model of T-cell lymphoma [21]. The development of noncovalent inhibitors is preferred due to the low risk of side-effects. Exosite binding and allosteric inhibitors also seem to be more promising [22].

### 3.4. Functions

The main function of MMPs is to degrade structural components of the ECM. However, MMP proteolysis can create space for cell migration, produce specific substrate-cleavage fragments with independent biological activity, regulate tissue architecture through effects on the ECM and intercellular junctions, and modify the activity of signaling molecules, both directly and indirectly [23]. Also, MMPs can affect cellular functions by regulating the ECM proteins with which the cells interact. The collagenases MMP-1, MMP-8, MMP-13, and MMP-14 are the only MMPs that can efficiently degrade the fibrillar collagens (types I, II, and III) in their triple-helical domains [24]. Cleavage by these enzymes renders the collagen molecules thermally unstable, so that they unwind to form gelatin, after which they can be degraded by other members of the MMP family such as the major gelatinases, MMP-2, and MMP-9. These two major gelatinases have several distinctive features. They can be distinguished by the fact that MMP-2 binds preferentially to TIMP-2, which is required for its activation, whereas MMP-9 is preferentially inhibited by TIMP-1 [25]. MMP substrates include peptide growth factors, tyrosine kinase receptors, cell adhesion molecules, cytokines, chemokines, as well as other MMPs and unrelated proteases. Furthermore, MMPs can participate in several other biological processes such as angiogenesis. Angiogenesis is a complex multistep process defined as the formation of new blood vessels from existing endothelial-lined vessels. This is distinct from the process of vasculogenesis in a way that the endothelial cells arise by proliferation from existing vessels rather than through differentiation from stem cells. Angiogenesis is an invasive process that requires proteolysis of the ECM and proliferation and migration of endothelial cells, as well as synthesis of new matrix components. Understanding the circumstances under which MMP activity is involved in the angiogenic phenotype has important implications for cancer therapy because angiogenesis is necessary for tumor growth and metastasis. It has been reported that MMP-2, MMP-9, and MT1-MMP released by endothelial cells are induced by vascular endothelial growth factor (VEFG) and basic fibroblast growth factor (bFGF), which are known to be potent mitogens and chemoattractants [26].

However, some MMP degradation products of ECM are antiangiogenic [27]. In summary, MMPs could have a double effect on angiogenesis. Programmed cell death, commonly termed apoptosis, is a very well ordered process by which unwanted, defective, or damaged cells are rapidly and selectively eliminated from the body. MMPs play an intriguing role in apoptosis, showing both apoptotic and antiapoptotic actions [28]. MMPs affect cell survival and proliferation both positively and negatively by regulating “survival signals”; these particular effects of MMPs may reflect the differences in MMP substrates involved in each response [29–31]. MMP-7 is able to release membrane-bound Fas ligand (FasL), which induces apoptosis of neighboring cells or decreases cancer cell apoptosis, depending on the system [32, 33]. Moreover, the cleavage of pro-heparin-binding epidermal growth factor (pro-HB-EGF) by MMP-7 leads to a biologically active HB-EGF that promotes cell survival by stimulating the erb-B4 receptor tyrosine kinase [34]. Other MMPs, such as MMP-11 and MMP-3, have a pro- or antiapoptotic effect depending on the circumstances [35–39]. The proapoptotic effect of MMP-2 and MMP-9 has been described during tissue remodeling and neoangiogenesis [40].

### 3.5. Analytical Methods

Several analytical methods can be used to measure MMPs, the choice of technique depending on the aim of the analysis. Here, we present the most widely used techniques. Zymography can be used to determine protease activity in both tissue homogenates and body fluids. Zymography and reverse zymography are simple, sensitive, quantifiable, and functional assays to analyze MMPs and TIMPs in biological samples [40]. Zymography is based on the following principles: retention of the substrate in the gel during electrophoresis, reversible inhibition of MMP activity by SDS during electrophoresis, and separation of MMP-TIMP complexes caused by SDS during electrophoresis. Zymography permits distinction of both proenzymes and active forms of MMPs based on their molecular weight [41, 42]. Several techniques can be used to determine MMP expression independently of zymogen or active form. First, western blot is a commonly used method in molecular biology to detect a target protein in a sample, containing a complex mixture of proteins, by using a polyclonal or monoclonal antibody specific to that protein. This technique is similar to zymography except that the gel does not contain substrate. Another technique is the enzyme-linked immunosorbent assay (ELISA). ELISA is a biochemical technique mainly used in immunology; the classical ELISA used for the detection of MMP is the sandwich ELISA. Specific antibodies to target protein are precoated onto 96-well plates. The test sample, body fluid or cell protein extract, is added to the wells [42]. Immunochemistry is another technique widely used for MMP detection. Serial sections are consecutively cut from formalin-fixed and paraffin-embedded tissue blocks using a microtome and are then transferred to adhesive-coated slides. This technique allows the determination of MMP localization and MMP producer cell type and a semiquantitative determination. Finally, polymerase chain reaction (PCR) can be used. As the name implies, this technique involves a chain reaction: one DNA molecule is used to produce two copies, then four, then eight, and so forth.

This continuous doubling is accomplished by specific proteins known as polymerases, enzymes that are able to string together individual DNA building blocks to form long molecular strands [42].

## 4. MMPs in Bladder Cancer

Some of the best candidates for a biological marker in bladder cancer are the MMPs, which have a well-known role in the degradation of connective tissue stroma and basement membranes (key barriers to tumor progression), promoting invasion, immune escape, and many other events.

In addition, recent data clearly challenge the classic dogma that MMPs promote metastasis only by modulating the remodeling of ECM. In fact, MMPs have also been attributed as an impact on tumor cell behavior in vivo as a consequence of their ability to cleave growth factors, cell surface receptors, cell adhesion molecules, and chemokines/cytokines [23, 29]. Furthermore, by cleaving proapoptotic factors, MMPs are able to produce an aggressive phenotype by generating apoptosis-resistant cells [43]. MMPs may also regulate angiogenesis in cancer, both positively through their ability to mobilize or activate proangiogenic factors [44] and negatively via generation of angiogenesis inhibitors, such as angiostatin and endostatin, which are cleaved from large protein precursors [45]. Consequently, several MMPs, in particular the gelatinases MMP-2 and MMP-9, have been widely studied. Conflicting results have been reported regarding the predictive value of MMP-2 and MMP-9 in bladder cancer. In this respect, it has to be borne in mind that studies may not be comparable since they may differ with respect to stage and grade of disease, blood (serum or plasma) and tissue sampling, and MMP levels in urine. Results in urine indicate that urine analyses are more useful for detection of progression and invasiveness than for early diagnosis, for which their sensitivity is even lower than that of urine cytology [46]. MMP-9 activities in urine samples have provided evidence that the use of MMP-9 as a screening or diagnostic marker should be explored, as its specificity appears comparable to that of urinary cytology [46, 47]. MMP-3 has no diagnostic significance and its prognostic utility is controversial [48, 49]. MMP-7 has shown prognostic relevance independent of the method of detection used [50]. MMP-14 is mostly expressed in stromal cells and can correlate with stage, grade, and prognosis [51]. The roles of MMP-10, MMP-11, MMP-13, and MMP-15 in bladder cancer have not been widely studied, but MMP-13 revealed higher expression in tumors with a higher stage and grade [52]. Decreased concentrations of TIMP-1 in plasma and increased concentrations in urine have been reported in bladder cancer [48, 53]. In the case of TIMP-2, two studies analyzing serum and plasma levels reported higher serum and plasma concentrations in controls than in bladder cancer patients [10, 54].

### 4.1. Tissue-Based Biomarkers

Davies et al. [55] reported that MMP-2 and MMP-9 activities quantified by gelatin zymography were correlated with tumor grade and invasion. Grignon et al. [56] showed that high levels of TIMP-2 expression are associated with poor outcome in cystectomy.

Kanayama et al. [51] evaluated MMP-2, TIMP-2, and MT1-MMP in bladder cancer tissue using RT-PCR and found MMP-2 and TIMP-2 expression levels to be strongly associated with tumor stage and prognosis; MT1-MMP expression, however, was directly correlated with distant metastasis but not with tumor invasion.

MMP-2 activity as determined by zymography has shown a strong correlation with tumor invasion. Papathoma et al. [57] reported that zymographic analysis of MMP-2 and MMP-9 showed a significant increase in levels with tumor grade and invasiveness, but the correlation between the levels of the two gelatinases with recurrence in NMI tumors was not significant. Ozdemir et al. [58] reported a strong correlation of basement membrane degradation with p53 inactivation and/or MDM2 overexpression in NMI urothelial carcinomas and suggested that MMP-9 plays a key role in the invasion step of NMI tumors. Kitagawa et al. [59] reported that MT1-MMP and MT2-MMP may play an important role in multifocality. Mohammad et al. [60], in a zymographic analysis in tissue samples, observed MMP-2 activity and MT2-MMP expression to be associated with tumor stage. Hara et al. [61] determined the expression levels of MMP-2, MMP-9, MT1-MMP, TIMP-1, and TIMP-2 messenger mRNA in UBC specimens using northern blot analysis. Results were evaluated in regard to tumor recurrence and indicated that MMP-9 and TIMP-2 were strongly expressed in tumors that displayed recurrence compared with those that did not. Vasala et al. [54] determined MMP-2 with a monoclonal antibody for MMP-2 by semiquantitative immunohistochemical staining of paraffin-embedded tissue. The results demonstrated that MMP-2 protein overexpression may be an independent prognostic biomarker for bladder cancer progression (Table 2).

### 4.2. Blood-Based Biomarkers

Blood is a good medium for analysis of markers for screening and detection purposes and can also be used for evaluation of prognostic markers. Advantages over tissue are that blood sampling is minimally invasive and provides information before surgery. However, blood sampling methods can strongly affect MMP measurements. Significantly lower MMP-1, MMP-2, and MMP-7 levels have been found in EDTA-treated plasma samples compared with citrate-stabilized or heparin-treated plasma. Also, MMP-8 and MMP-9 showed significantly higher serum concentrations when collected in tubes with clot activator than when collected in tubes without such activator. Gohji et al. reported the prognostic significance of serum MMPs and TIMPs and the value of imbalance between serum MMP-2 and its inhibitor as a predictor of recurrence [4, 62–64]. Angulo et al. [65] used RT-PCR to analyze circulating blood cells for MMP-2, MMP-9, and TIMP-2, and revealed that MMP-9 has a higher ability to distinguish metastatic disease but that MMP-2 is better at discriminating levels of tumor invasion [8]. Szarvas et al. [66] analyzed MMP-7 in serum using ELISA and concluded that circulating MMP-7 levels may help to identify bladder cancer patients at high risk of disease progression. Vasala et al. [10, 54], using ELISA, determined that high serum levels of circulating pro-MMP-2 and TIMP-2 are associated with a better clinical course; moreover, total pro-MMP-2 was found to be an independent prognostic marker of bladder cancer progression. Tasci et al. [67] analyzed genomic DNA extracted from peripheral blood lymphocytes. Their results suggested that the MMP-1 promoter polymorphism might be linked to susceptibility for bladder cancer (Table 2).

### 4.3. Urine Biomarkers

In patients with bladder cancer, urine is a particularly useful medium to detect tumor markers owing to its enhanced potential to contain high concentrations of tumor-derived proteins. Recent data have also shown that elevated urinary MMP levels and activity correlate with the presence of different cancers located outside the urinary tract [13].

Offersen et al. found MMP-9 [68] measured in urine from bladder cancer patients to be a strong independent prognostic marker of poor survival. Szarvas et al. [69] determined the presence of MMP-7 in the urine of patients with bladder cancer using immunoprecipitation followed by western blot analysis and observed that MMP-7 could help to detect metastatic disease. Hoque et al. [70] examined urine sediment DNA for aberrant methylation of nine genes, including TIMP-3, by quantitative fluorogenic RT-PCR. Results suggested that TIMP-3 promoter methylation could be a clinically applicable marker for bladder cancer progression. Eissa et al. obtained urine samples and used urine sediment for cytology and the supernatant for estimation of MMPs and TIMP-2 by ELISA and gelatin zymography. Combined testing of cytology with these methods improved the sensitivity of bladder cancer detection, even in superficial and low-grade tumors [71]. Holten-Andersen et al. [72] assessed the potential use of TIMP-1 levels in plasma and urine. The results revealed that the measurement of TIMP-1 in plasma and/or urine was apparently not useful for the identification of bladder cancer. Finally, Di Carlo et al. used zymography to analyze MMP-2 and MMP-9 in urine [73] and observed that the urinary values of these two biomarkers correlated with the increase in MMP-9 lytic activity in high-grade and advanced-stage bladder cancer (Table 2).

## 5. Conclusions

Bladder cancer represents the most common tumor of the urinary tract. The prognosis of the tumors diagnosed at an early stage is excellent, emphasizing the importance of early diagnosis. Measurements of MMPs in urine, especially MMP-2 and MMP-9, show a superior diagnostic performance compared with cytology but are less sensitive than determination of other diagnostic markers such as NMP-22. MMPs could be useful when used in combination with other methods rather than alone. The need for lifelong surveillance makes bladder cancer one of the most expensive cancers; thus MMPs are needed to allow the identification of patients at risk of recurrence. Urinary MMP-9 levels, serum MMP-7 levels, and tissue levels of MMP-2, MMP-7, and MMP-14 have been identified as prognostic markers of bladder cancer.

## Figures and Tables

**Table 1:** Human matrix metalloproteinases (MMPs).

No.	MMP	MMP class	Enzyme	Molecular weight (kDa)	Substrates
1	MMP-1	Collagenases	Collagenase-1	57^∗^ 47 A	Collagens (I, II, III, VII, and X), proteoglycans, entactin, ovostatin, MMP-2, MMP-9
2	MMP-8	Collagenases	Collagenase-2/neutrophil collagenase	85^∗^ 64 A	Collagens (I, II, III, VII, VIII, and X), fibronectin, proteoglycans
3	MMP-13	Collagenases	Collagenase-3	60^∗^ 48 A	Collagens (I, II, III, VII, VIII, and X), tenascin, plasminogen, aggrecan, fibronectin, osteonectin, MMP-9
4	MMP-18	Collagenases	Collagenase-4	53^∗^ 51 A	Type I collagen
5	MMP-2	Gelatinases	Gelatinase-A	72^∗^ 66 A	Gelatin, collagen (IV, V, VII VI, IX, and X), elastin, fibronectin
6	MMP-9	Gelatinases	Gelatinase-A	92^∗^ 86 A	Collagens (IV, V, VII, X, and XIV), gelatin, entactin, elastin, fibronectin, osteonectin, plasminogen, proteoglycans
7	MMP-3	Stromelysins	Stromelysin-I	60^∗^ 52 A	Collagens (IV, V, and IX), gelatin, aggrecan, laminin, elastin, casein, osteonectin, fibronectin, ovostatin, entactin, plasminogen
8	MMP-10	Stromelysins	Stromelysin-2	53^∗^ 47 A	Collagens (I, II, IV, and V), gelatin, casein, elastin, fibronectin
9	MMP-11	Stromelysins	Stromelysin-2	60^∗^ 47 A	Collagens (IV, V, IX, and X), laminin, elastin, fibronectin, casein, proteoglycans
10	MMP-17	Stromelysins	Homology tostromelysin-2	65^∗^ 63 A	Pro-MMP2, fibrin/fibrinogen, gelatin
11	MMP-7	Matrisylins	Matrisylin	28^∗^ 19 A	Collagen IV, gelatin, fibronectin, laminin, elastin, casein, transferrin
12	MMP-26	Matrisylins	Matrisylin-2	29	Collagen IV, fibronectin, fibrinogen, gelatin, pro-MMP9
13	MMP-14	MT-MMP (membrane type-MMP)	MT1-MMP	66^∗^ 54 A	Collagens (I, II, III), gelatin, fibronectin, laminin, vitronectin, entactin, pro-MMP-2
14	MMP-15	MT-MMP	MT2-MMP	76	Fibronectin, gelatin, vitronectin, entactin, laminin, pro-MMP-2
15	MMP-16	MT-MMP	MT3-MMP	65^∗^ 63 A	Collagen III, gelatin, casein, fibronectin, pro-MMP-2
16	MMP-17	MT-MMP	MT4-MMP	65^∗^ 63 A	Pro-MMP-2, fibrinogen, gelatin
17	MMP-24	MT-MMP	MT5-MMP	73	Fibronectin, pro-MMP-2, proteoglycans, gelatin
18	MMP-25	MT-MMP	MT6-MMP	62	Pro-MMP-2, pro-MMP-9, collagen IV, gelatin, fibronectin, proteinase A
19	MMP-12	Other enzymes	Macrophage metalloelastase	54^∗^ 45 A	Collagen IV, gelatin, elastin, casein, fibronectin, vitronectin, laminin, entactin, fibrin/fibrinogen
20	MMP-19	Other enzymes	RASI I	59	Collagen (I, IV) gelatin, fibronectin, laminin
21	MMP-20	Other enzymes	Enamelysin	56	Amelogenin, aggrecan
22	MMP-21	Other enzymes		65	
23	MMP-22	Other enzymes		58^∗^ 53 A	
24	MMP-23	Other enzymes		44	Gelatin
25	MMP-27	Other enzymes		59	
26	MMP-28	Other enzymes	Epilysin	59	

^∗^Zymogen molecular weight; A: active form molecular weight.

**Table 2:** Correlation between MMP levels and clinicopathologic parameters. NA: not available; T: tissue; B: blood; U: urine.

Author	B/T/U	Pat/Con	Method	Expres.tm	Stage	Grade	Recurrence	Prognosis	MMP-
Gohji et al. [4]	B	146/52	Enzyme assay	Increased	NA	NA	Yes	NA	MMP-2 MMP-3
Angulo et al. [65]	B	31/11	RT-PCR	Increased	Increased	NA	NA	NA	MMP-2 MMP-9 TIMP-2
Szarvas et al. [50]	B	97/22	Enzyme assay	Increased	NA	NA	NA	Yes	MMP-7
Vasala et al. [10]	B	84/—	ELISA	Decreased	NA	NA	NA	Yes	pro-MMP-2 MMP-2 TIMP-2
Tasci et al. [67]	B	102/94	PCR lymphocytes	Increased	Increased	Increased	NA	NA	MMP-1
Offersen et al. [68]	U	188/—	Immunocapture activity assay	Increased	Increased	Increased	Yes	Yes	MMP-9
Szarvas et al. [69]	U	132/96	Enzyme assay	Increased	NA	NA	NA	Yes	MMP-7
Hoque et al. [70]	U	175	RT-PCR	Methylation	Increased	NA	NA	Yes	TIMP-3
Eissa et al. [71]	U	136/60	ELISA Gelatin zymography	Increased	NA	Increased	NA	NA	MMP-2 MMP-9 TIMP-2
Di Carlo et al. [73]	U	25/—	Gelatin zymography	Increased	Increased	Increased	NA	NA	MMP-9
Davies et al. [55]	T	42/7	In situ hybrid	Increased	Increased	NA	NA	Yes	MMP-2 MMP-9
Grignon et al. [56]	T	42/—	IHQ	Increased	Increased	NA	NA	Yes	TIMP-2
Kanayama et al. [51]	T	41/—	RT-PCR	Increased	Increased	NA	NA	Yes	MMP-2 TIMP-2
Papathoma et al. [57]	T		Gelatin zymography IHQ	NA	NA	NA	NA	Yes	MMP-2 MMP-9
Hara et al. [61]	T	51	Northern blot analysis	NA	Increased	Increased	Yes	NA	MMP-9 TIMP-2
